# The absence of proximal contact point on periodontal parameters of teeth moved into extraction sites

**DOI:** 10.1590/0103-6440202204330

**Published:** 2022-06-24

**Authors:** Jocimara Domiciano Fartes de Almeida Campos, Marcio José da Silva Campos, Paula Liparini Caetano, Cassiano Kuchenbecker Rösing, Robert Willer Farinazzo Vitral

**Affiliations:** 1 Department of Periodontology- Estácio Juiz de Fora University Center, Juiz de Fora - MG, Brasil; 2 Department of Orthodontics- Federal University of Juiz de Fora, Juiz de Fora - MG, Brasil; 3 Department of Periodontology- Federal University of Rio Grande do Sul, Porto Alegre - RS, Brasil

**Keywords:** Periodontics, Orthodontic space closure, Tooth extraction, Dental occlusion

## Abstract

The aim of study was to evaluate periodontal conditions of upper canines and second premolars with and without proximal contact of individuals undergoing orthodontic treatment associated to extractions of the upper first premolars. The study selected upper canines and premolars of individuals undergoing orthodontic treatment without extractions (30 hemiarches - control group), or with extraction of the upper first premolars and whose canines and second premolars had interproximal contact (16 hemiarches - group 1) or diastema (17 hemiarches - group 2). Clinical (plaque index, probing depth, gingival bleeding index, height of the gingival margin, clinical attachment loss and gingival clefts) and radiographic (crest height, bone height and bone-crest discrepancy) parameters of the distal surfaces of canines and mesial surfaces of premolars were evaluated. Group 1 had worse results when compared to the control group for the levels of plaque in canines and premolars and for probing depth in canines (distal and mean) and in premolars (lingual and mean), as well as increasing tendency of clinical attachment loss (lingual and mean) in premolars. Plaque level in canines in group 1 was also significantly higher than in group 2. There was no difference between group 2 and the control group. The lack of proximal contact between canines and second premolars did not significantly affect their periodontal characteristics.

## Resumo

A extração de dentes para obtenção de espaço nos arcos dentários é uma estratégia rotineiramente utilizada em Ortodontia, porém a movimentação de dentes para os locais de extrações pode estar associada à falhas na estabilidade dos dentes e abertura do ponto de contato, gerando prejuízo aos tecidos periodontais.

Objetivo: Avaliar a condição periodontal de caninos e segundos pré-molares superiores com e sem ponto de contato entre si de indivíduos submetidos ao tratamento ortodôntico associado à extração dos primeiros pré-molares superiores. Foram selecionados caninos, primeiros e segundos pré-molares superiores de indivíduos submetidos ao tratamento ortodôntico sem extração (30 hemiarcos - grupo controle) ou com extração dos primeiros pré-molares superiores cujos caninos e os segundos pré-molares apresentavam contato interproximal (16 hemiarcos - grupo 1) ou diastema (17 hemiarcos - grupo 2). Nas superfícies distal dos caninos e mesial dos pré-molares foram avaliados parâmetros clínicos e radiográficos para determinar o efeito da movimentação dos dentes para o local de extração e da ausência de ponto de contato interproximal nos tecidos periodontais. Os grupos não apresentaram diferença significativa para a idade e o tempo de pós-tratamento. O grupo 1 apresentou valores mais desfavoráveis em relação ao grupo controle para o índice de placa em caninos e pré-molares e para a profundidade de sondagem em caninos (distal e média) e em pré-molares (lingual e média), além de uma tendência de maior perda de inserção clínica (lingual e média) nos pré-molares. O índice de placa nos caninos do grupo 1 também foi significativamente maior do que no grupo 2. Os grupos 2 e controle não apresentaram diferença significativa. A ausência de ponto de contato entre os caninos e os segundos pré-molares superiores não afetou significativamente a situação periodontal desses dentes.

## Introduction

Steady and well-located contact points on proximal surfaces are considered essential for periodontal health, as they avoid food impaction during mastication, which facilitates periodontal diseases ^1^ ^,^ ^2^ by compromising the attachment of tooth to the junctional epithelium ^3^.

The lack of proximal contact between dental surfaces can lead to local inflammation and eventual loss of tooth supporting tissues, mainly due food impaction and plaque retention ^1^ ^,^ ^2^ ^,^ ^4^. It can also cause acute papillary gingivitis, gingival abscess, increased probing depth and interproximal clinical attachment loss ^2^, having a negative impact on patient satisfaction after orthodontic treatment ^5^.

Teeth extraction to gain space for proper teeth alignment is a routine strategy in orthodontic treatment, with the first premolars being the most suitable for extraction ^6^. Teeth movement into extraction sites was linked to reduction of the interproximal bone height ^6^ ^,^ ^7^. Furthermore, teeth moved into extraction spaces can exhibit stability failure, which leads to interproximal contact loss ^8^. This abnormal relation between proximal surfaces can cause food impaction and retention during mastication ^2^ ^,^ ^5^ ^,^ ^9^.

Previous studies ^6^ ^,^ ^10^ ^,^ ^11^ ^,^ ^12^ have already analyzed periodontal conditions of teeth moved into extraction spaces, but only Artun and Osterberg ^11^ considered the influence of whether or not there is proximal contact between teeth moved into extraction spaces in their periodontal parameters.

It should be noted that there are few studies on the impacts of missing proximal contact after orthodontic treatment of periodontal tissues, and they do not support the possible effects of this relevant clinical condition on patients’ periodontal health and well being in a conclusive manner.

The aim of study was to evaluate periodontal conditions of upper canines and second premolars with and without proximal contact of individuals who underwent orthodontic treatment associated to extractions of the upper first premolars.

## Methods

The Human Subjects Ethics Board of Federal University of Juiz de Fora (number 1.945.940) approved this study and all the subjects participated as volunteers and signed a written informed consent form accepting to be part of the research. The sample size was initially calculated based on continuous measurement for two samples ^12^, using t-statistic with a non-centrality parameter and a significance of α = 0.05, error type II β = 0.20 and standard deviation of S=0.4. The sample size calculation indicated the need of 12 hemiarches on each group to reject the null hypothesis that group populations are the same, considering an effect size of 1,250 and a statistical power of 0.8.

This cross-sectional study selected subjects who finished orthodontic treatment with edgewise appliances in which the use of upper removable retainers had been suspended for at least one year and the treatment of the upper arch did not include any extractions or only first premolar extraction. Subjects should have complete permanent dentition prior to treatment (except for permanent upper second and third molars), completely erupted upper canines and premolars without vertical bone loss or rotation of more than 10^o^. The sample did not include subjects with clinical and/or radiographic evidence of periodontal diseases, over contoured proximal restoration, or fixed retention in upper canines, first or second premolars.

The control group was composed by canines, first and second premolars of 30 upper dental hemiarches of 16 individuals (10 females and 6 males) treated without extractions, with mandatory proximal contact between these teeth. In subjects who underwent orthodontic treatment with extraction of the upper first premolar, teeth adjacent to the extraction spaces (upper canine and second premolars) with proximal contact were put in group 1 (10 individuals - 7 females and 3 males - 16 hemiarches) and teeth adjacent to the extraction spaces without proximal contact were put in group 2 (9 individuals - 4 females and 5 males - 17 hemiarches). The absence of proximal contact was diagnosed by passing an unwaxed dental tape with no resistance between the teeth ^9^. As the number of hemiarches evaluated was higher than the one defined beforehand on the sample calculation (N= 36), statistical power was raised to 0.95.

### Clinical evaluation

This study measured plaque index ^13^ and probing depth ^14^ on buccal, lingual, mesial and distal surfaces and gingival bleeding index ^15^, height of the gingival margin (distance from gingival margin and the cement-enamel junction - positive values indicate gingival recession) ^14^ and clinical attachment loss (distance from the cement-enamel junction and the bottom of the gingival sulcus) on buccal and lingual surfaces of evaluated canines and premolars. The Williams-type periodontal probe (Trinity, São Paulo, Brazil) was used to perform the clinical evaluation.

It was also evaluated whether or not there were gingival clefts on buccal and lingual surfaces of the alveolar ridge in extraction spaces of groups 1 and 2. Every interproximal tissue invagination with at least 1mm depth was considered a gingival cleft ^16^.

Clinical and radiographic evaluations of each subject were conducted by a single evaluator, a periodontist with 10 years of experience (J.D.F.A.C.), on the same appointment, with a minimum one hour interval after ingesting food and liquids (except for water), smoking cigarettes or any oral hygiene procedures.

### Radiographic evaluation

All teeth included in the three groups were subjected to interproximal digital radiography with Kwik-Bite phosphor plate holder. The center of the sensor (40x30mm) was positioned in the mesiodistal center of the upper first premolar in the control group and in the center of the second premolar in groups 1 and 2, and the central axis of the x-ray beam was also directed to the same point.

The interproximal alveolar bone was evaluated on the distal faces of canines (three groups) and mesial faces of the first (control group) and second premolars (three groups). Only the proximal surfaces adjacent do extraction spaces and the mesial surfaces of the first premolars, as they relate to the distal surfaces of canines, were evaluated. A perpendicular line was drawn along the dental axis, through the cement-enamel junction of the proximal surface being evaluated (CEJ line) using the software Image J 1,46R (National Institutes of Health, USA). From the CEJ line, the distances until the most coronal point of the alveolar bone crest (crest height) and to the most coronal point of the alveolar bone where the periodontal ligament remains even (bone height) were measured ^10^. Moreover, the difference between these two distances was calculated (bone-crest discrepancy).

### Food impaction

Subjects without proximal contact (group 2) were questioned about food impaction on interproximal spaces, how frequently they experienced food retention in these spaces, the pain caused by such impaction, the difficulty of removing food remains and the kinds of food that usually get retained in there.

### Calibration and statistics

To measure the mean error of the researcher responsible for the evaluations (J.D.F.A.C.), a comparison was made of the values of all variables of 30 teeth obtained in two different moments, with a 15-day interval, and the reproducibility of these measures was evaluated using the Intraclass Correlation Coefficient (ICC) or Cohen’s Kappa Coefficient.

The difference between the groups for the gingival bleeding index and the presence of gingival clefts was evaluated using Pearson’s chi-squared test. The analysis of variance (ANOVA) was used for other variables, and Tukey’s HSD post hoc test was used when significant differences were found. For the second premolars (groups 1 and 2) to be compared to the first and second premolars of the control group, the first premolars were considered a specific group during tests. The significance level used was α=0,05 and the data was processed with the SPSS Statistics 20.0.0 (SPSS, Chicago, IL, USA) software.

## Results

The values obtained for the error of the method showed satisfactory or excellent reproducibility for ICC (< 0.430) and reasonable for Cohen’s Kappa Coefficient (< 0.262).

The age at the beginning of treatment for control group, group 1 and group 2 were 27.80, 29.18 and 29.53 years and the retention period were 4.62, 5.0 and 6.23 years, respectively. There were not significantly different between groups. For all clinical and radiographic parameters, the first premolars of the control group had no significant difference from the second premolars of the same group.

Group 1 had the highest levels of plaque and percentage of sites with plaque (table 1). They were significantly higher levels of plaque than group 2 and control group in canines and then control group in first and second premolars. Positive scores of gingival bleeding were registered between 16.1 and 32.3% of surfaces evaluated, with no significant difference between the groups (table 2).

**Table 1:** Clinical evaluation (plaque index) in the canines and premolars of control group, group 1 and group 2.

	Control group				Group 1				Group 2				p-valor*
	N	Mean	SD	%	N	Mean	SD	%	N	Mean	SD	%	
Plaque index													
Canines	30	0.48^a^	0.508	46,6	16	1.31^ab^	0.793	88,2	17	0.64^b^	1.057	29,4	0.003
1st premolar - CG	30	0.51^a^	0.553	46,6	16	1.31^ab^	0.793	88,2	17	0.68	1.038	41,2	0.002
2nd premolar - CG	30	0.51^b^	0.553	46,6									

CG - Control group; N - number of teeth; SD - Standard deviation; % - percentage of sites with plaque (index 1, 2 and 3). * - Analysis of variance (ANOVA) followed by Tukey’s HSD post hoc test (the same letter indicate significant difference)

**Table 2:** Gingival bleeding index in the canines and premolars of control group, group 1 and group 2.

Teeth/face	Control group			Group 1			Group 2			p-valor*
	N	( - )	( + )	N	( - )	( + )	N	( - )	( + )	
*Canine*	30	67.7%	32.3%	16	81.3%	18.8%	17	82.4%	17.6%	0.429
*1st premolar - CG*	30	71.0%	29.0%	16	75.0%	25.0%	17	76.5%	23.5%	0.977
*2nd premolar - CG*	30	74.2%	25.8%							

N - number of teeth; (-) - negative; (+) - positive; CG - control group; * - Chi-square test.

Regarding the height of the gingival margin (table 3), group 1 had the lowest scores in all surfaces and teeth, except on the lingual surface of the second premolars (0.01mm higher than group 2), which indicates the gingival margin to be positioned more to the coronal direction, with no significant difference in any of the comparisons. Gingival recession was identified between the vestibular and lingual faces of teeth in 11.8% of the control group, 13.6% of group 1 and 14.7% of group 2.

**Table 3:** Clinical evaluation (height of the gingival margin, probing depth and clinical attachment loss) in the canines and premolars of control group, group 1 and group 2.

	Control group			Group 1			Group 2			p-valor*
	N	Mean	SD	N	Mean	SD	N	Mean	SD	
Plaque index										
*Canines*	30	0.48^a^	0.508	16	1.31^ab^	0.793	17	0.64^b^	1.057	0.003
*1st premolar - CG*	30	0.51^a^	0.553	16	1.31^ab^	0.793	17	0.68	1.038	0.002
*2nd premolar - CG*	30	0.51^b^	0.553							
Height of the gingival margin										
*Canines*	30	-0.55	0.799	16	-0.78	0.893	17	-0.41	0.814	0.436
*1st premolar - CG*	30	-0.37	0.982	16	-0.69	1.181	17	-0.50	0.684	0.738
*2nd premolar - CG*	30	-0.55	0.916							
Probing depth										
*Canines*	30	1.39	0.417	16	1.70	0.518	17	1.64	0.424	0.048^+^
*1st premolar - CG*	30	1.55^a^	0.444	16	1.98^a^	0.451	17	1.69	0.410	0.018
*2nd premolar - CG*	30	1.66	0.435							
Clinical attachment loss										
*Canines*	30	0.31	0.681	16	0.50	0.912	17	0.82	1.014	0.141
*1st premolar - CG*	30	0.38	0.612	16	0.93	1.123	17	0.79	0.751	0.037^†^
*2nd premolar - CG*	30	0.40	0.626							

CG - Control group; N - number of teeth; SD - Standard deviation; * - Analysis of variance (ANOVA) followed by Tukey’s HSD post hoc test (the same letter indicate significant difference); + - The post hoc test indicated a p-value of 0.072 between the control group and group 1. † - The post hoc test indicated a p-value of 0.084 between the 2nd premolars of group 1 and the 1st premolars of the control group.

For probing depth evaluation, group 1 had the highest means in almost every location in which the measurements were made (table 3). The mean value of the distal surface of canines was significantly higher than the control group. The average of all surfaces of canines was significantly higher in group 1 when ANOVA test was used. However, when two groups were compared, p-values were higher than the significance level used in the study. The second premolars of group 1 had significantly higher values than the first and second premolars of the control group on the lingual surface and the the first premolars of the control group on average.

Table 3 describes the results of clinical attachment loss. Group 2 had the highest average values, but with no significant difference to other groups. As for premolars, the ANOVA revealed a difference between groups for values on the lingual surface and on the average of all surfaces, although multiple comparisons between pairs of groups had no significant difference even though some p-values were close to the significance level used for this study (0.054, 0.059 and 0.084).

Interproximal spaces between canines and premolars of group 1 and group 2 show gingival clefts at 12.5% and 23.5% respectively, with no difference between groups. The gingival clefts were always present on the buccal and lingual surfaces.

Table 4 shows the results of radiographic evaluations. The canines of group 1 had the highest values for bone and crest heights, whereas group 2 had the highest discrepancy between these two values, although there was no significant difference in any of the comparisons. Group 1 also had the highest means for radiographic variables in the mesial region of premolars, including bone-crest discrepancy, with no difference between the means of the groups.

Most of the subjects with interproximal spaces without proximal contact (group 2) reported food impaction in these interproximal spaces, with low (33.3%) or high (44.5%) frequency, although this was not linked to pain or difficulty in removing food for any of them. When there is no proximal contact, 66.7% of subjects reported retention of all kinds of food consistency, with fibrous foods being specifically linked to food impaction by only 11.1% of subjects.

**Table 4:** Radiographic evaluation - Crest height and bone height.

		Control group			Group 1			Group 2			p-valor*
		N	Mean	SD	N	Mean	SD	N	Mean	SD	
*Canine*											
*Distal bone height*		30	1.40	0.705	16	1.66	0.570	17	1.57	0.577	0.510
*Distal crest height*		30	="2">1.15	0.635	16	1.37	0.473	17	1.15	0.454	0.528
*Bone-crest discrepancy*		30	0.25	0.230	16	0.29	0.299	17	0.41	0.325	0.246
*Premolar*											
*Mesial bone height*	1st premolar - CG	30	1.38	0.570	16	1.79	0.870	17	1.28	0.466	0.164
	2nd premolar - CG	30	1.25	0.722							
*Mesial crest height*	1st premolar - CG	30	1.09	0.587	16	1.28	0.584	17	0.95	0.407	0.479
	2nd premolar - CG	30	1.01	0.594							
*Bone-crest discrepancy*	1st premolar - CG	30	0.29	0.202	16	0.50	0.460	17	0.33	0.182	0.078
	2nd premolar - CG	30	0.24	0.249							

N - number of teeth; SD - Standard deviation. * - Analysis of variance (ANOVA).

## Discussion

This study aimed to verify the impacts of orthodontic movement of upper second premolars into extraction spaces and of the presence of proximal contact between these teeth over periodontal parameters. The relevance of this kind of study lies in the fact that the lack of proximal contact not only represents potential discomfort and dissatisfaction to patients ^5^, but can also cause irreversible damage to tooth-supporting tissues ^1^ ^,^ ^2^ ^,^ ^4^ ^,^ ^17^. In orthodontic treatment, premolar extraction enhances the chances of irregular proximal contact.

The indication for dental extractions in order to obtain space in dental arches has been frequent in orthodontic treatment. This therapeutic decision has been linked to local periodontal alterations due to teeth movement into extraction spaces ^6^ ^,^ ^7^ and/or to food impaction and retention in interproximal spaces after proximal contact loss ^5^ ^,^ ^9^.

This observational cross-sectional study compared subjects who underwent orthodontic treatment without extraction to those who had their upper first premolars extracted. To determine the periodontal effects of the lack of proximal contact in teeth moved into extraction spaces, two control groups were needed: a positive control, formed by teeth moved into extraction spaces with proximal contact (group 1) and a negative control, with no extraction and no proximal contact loss (control group).

Unlike previous studies that compared spaces between canines and second premolars to interproximal regions of canines and lateral incisors ^11^, premolars to molars ^18^ or canines to lower premolars ^6^, this study was based on the comparison between homologous teeth, as recommended by other studies ^7^ ^,^ ^10^ ^,^ ^12^. It also compared second premolars of groups 1 and 2 to first premolars of the control group, allowing the comparison of teeth and interproximal areas with similar morphology and anatomic location, leading to biologically reliable results.

A sample calculation was made for this study and resulted in a minimum of 12 hemiarches on each group, which was exceeded by the sample. This guarantees internal validation with the possibility of extrapolation to similar groups - young subjects who underwent orthodontic treatment with upper premolar extraction. Other studies published on this theme also had a low number of subjects ^1^ ^,^ ^6^ ^,^ ^12^ ^,^ ^16^.

The height of the alveolar crest reflected the most coronal portion of the bone septum adjacent to the interproximal space, while the height of the proximal bone referred to the most coronal bone portion where bone, periodontal ligament and tooth have a normal relation ^19^. Teeth moved into extraction spaces with proximal contact (group 1) had the proximal bone and the bone crest further from the cement-enamel junction, but there was no significant difference between the groups. In a similar manner, Lombardo et al. ^12^ reported nonsignificant bone reduction related to the amount of teeth movement into extraction spaces for immediate post treatment.

Besides measuring the alveolar crest and proximal bone heights individually, the discrepancy between them was also determined, since its increase can indicate intraossous defects on the teeth’s proximal surfaces ^19^. The highest values were found on the mesial faces of canines in group 2 and on the distal faces of second premolars in group 1. Although the mean differences did not exceed 0.26mm and were not significantly higher, the surfaces of teeth moved into extraction spaces had values of bone-crest discrepancy between 20 and 108% higher than their respective controls.

In cases of first pre-molar extractions, a steeper reduction of the alveolar bone is expected on the distal faces of canines than on the mesial faces of the second premolars, as the canines are usually subjected to more movement to the extraction sites than the premolars ^7^ ^,^ ^10^. Although this relation was observed between canines and second premolars of group 2, in the control group not only the proximal bone but also the bone crest was positioned more apically on the distal faces of canines in relation to the mesial faces of the second premolars, which suggests that the bone height difference occurs despite the movement of teeth into extraction sites.

The mean probing depth presented by the second premolars of group 1 was significantly higher than in control group, with the same tendency towards the clinical attachment loss and alveolar bone measurements, which suggests that the alteration of these clinical periodontal parameters is linked to the apical position of the adjacent alveolar bone ^19^. Previous studies reported increased probing depth ^11^ and clinical attachment loss ^10^ ^,^ ^11^, besides a reduction on the alveolar bone crest height in teeth adjacent to the extraction sites ^6^ ^,^ ^7^ ^,^ ^10^ ^,^ ^20^. However, the authors used fully banded orthodontic appliances ^7^ ^,^ ^10^ ^,^ ^11^, included individuals without orthodontic treatment in the control group ^7^ ^,^ ^10^ or compared non-homologous interproximal regions ^6^ ^,^ ^11^ ^,^ ^20^, which does not allow for a comparison with this study.

Nowadays studies that evaluated individuals whose all teeth were banded ^7^ ^,^ ^10^ ^,^ ^11^ have limited clinical applicability, since orthodontic bands were linked to gingival inflammation, gingival bleeding and increased probing depth ^21^ on teeth in which they are inserted. These alterations could be limited to the period of usage or could endure after the treatment was over ^21^.

After orthodontic treatment associated to teeth extraction, the teeth that were moved into extraction sites can exhibit stability failure on the position obtained by the end of treatment, resulting in proximal contact loss in approximately 30% of cases ^8^. This abnormal relation between proximal surfaces can result in food impaction and retention during mastication ^2^ ^,^ ^5^ ^,^ ^9^, therefore making it important to determine the individuals’ perspectives on this subject. In this study, food impaction was reported by almost 80% of individuals who lacked the contact point between the canine and the second premolar - which is higher than previously described for tooth/tooth (5%) ^1^ and tooth/implant interface (63%) ^9^. According to the individuals’ reports, every kind of food gets retained in these interproximal spaces that lack a contact point, with a discreet prevalence of fibrous foods, although this retention is not linked to pain or removal difficulty. These person-centered outcomes are important for an evidence-based dentistry practice, in which the patients’ perceptions, preferences and beliefs must be taken into consideration ^22^.

The absence of a contact point and subsequent food impaction have been related to clinical ^1^ ^,^ ^2^ and radiographic ^23^ periodontal changes. Contrary to the tendency described, when comparing interproximal spaces that differ only by the contact point (groups 1 and 2), teeth without interproximal contact point have more favorable results in all parameters that were evaluated (excepts for the clinical attachment loss of canines) with a significant difference only for the values of dental plaque in canines. Smaller plaque accumulation and the absence of contact point between the proximal faces was described previously ^2^ and can be explained by easier hygiene care allowed by the open interproximal contact point, despite the higher frequency of food impaction.

Although routine food impaction was described by 88% of individuals with open interproximal contact point and this condition is linked to intra osseous defects in adjacent dental faces ^23^, the lack of the contact point did not influence in a significant manner the bone-crest discrepancy, with the highest mean values being identified between teeth with (second premolars) and without (canines) contact point.

In this study, the clinical attachment loss was the only parameter negatively influenced by the lack of the contact point, with this value being 0.32mm (64%) higher in canines without the contact point, but with no statistical significance. Jernberg et al. ^2^ reported a similar score (0.48mm) for the difference of clinical attachment loss between contralateral interproximal spaces with and without contact point, but in individuals who did not undergo orthodontic treatment. Although the association between the absence of the contact point and clinical periodontal changes was suggested ^2^, these values must be interpreted with care, as an attachment loss lower than 0.5mm should not be considered clinically relevant ^11^.

Gingival clefts are considered a side effect of extraction space closure ^16^ ^,^ ^24^ ^,^ ^25^, and were observed in 18.2% of interproximal spaces of teeth moved into extraction sites, with no difference between the spaces with and without contact point. Previous studies reported gingival clefts in 4% ^11^, 10% ^16^ and even 93.3% ^25^ of extraction space closure in the maxilla. Although these clefts were associated to the reduction of interproximal bone levels ^24^, this relation was not set in this study as the group with the highest incidence of gingival clefts (group 2) did not show less favorable results for bone levels, neither for probing depth, clinical attachment loss and plaque accumulation, clinical parameters associated to gingival clefts ^11^.

Acknowledging the fact that this study has advantages and limitations, among the advantages, the comparison of individuals submitted to orthodontic treatment without the interference of a previously set investigation protocol makes the results more to resemble the usual patients of orthodontic treatment. It is also important to highlight the limited number of individuals. Regarding the limitations of the study, its cross-sectional type does not allow for causal inference. There is also not a possibility of a longitudinal observation of the individuals’ plaque control, so the results are limited to associations. Another limitation to be considered is that only the probing depth was evaluated in the distal site of canines and mesial site of premolars, and no clinical attachment loss was determined due to the difficult visualization of the cement-enamel junction in these places.

Overall, there were no relevant periodontal alterations due to the existence or not of a contact point between canines and second premolars, with orthodontic movement of such teeth into extraction sites being the main cause for these outcomes. The lack of a contact point must not be considered a causal factor for periodontal diseases, but a modifier of the periodontal condition as it compromises tooth attachment to the junctional epithelium ^3^ and facilitates the occurrence of etiologic agents linked to these problems ^20^.

## Conclusion

The lack of a contact point between upper canines and second premolars had no significant impact on the periodontal conditions of these teeth.
